# A Low-Energy Patella Vertical Dislocation in an Adolescent: A Case Report

**DOI:** 10.5704/MOJ.2307.013

**Published:** 2023-07

**Authors:** A Japamadisaw, TW Martanto, KD Hernugrahanto

**Affiliations:** Department of Orthopaedic and Traumatology, Universitas Airlangga, Surabaya, Indonesia

**Keywords:** closed reduction, general laxity, low-energy injury, vertical patella dislocation

## Abstract

Intra-articular dislocation of the patella is considered a rare case where it was reported that limited cases are existing in the literature and the exact mechanism of the injury is still undetermined. Patellar dislocation is divided into extra-articular and intra-articular dislocation. We report a patient with vertical dislocation of the patella caused by a low-energy injury that is very rare according to the previous study. The patient came with a deformity, skin tenting, and pain with pressure on the superior and medial sides of the patella. During the physical examination, a deformation of skin tenting was observed with the characteristic of a “dorsal-fin” appearance over the laterally displaced patella. This paper will discuss the dislocation of the patella, which can be further classified into extra-articular and intra-articular. Vertical patellar dislocation most commonly occurred in adolescence. The outcome was considered satisfactory, and this case provides further knowledge of the mode of injury of vertical dislocation and also the possible risk factors.

## Introduction

The incidence of vertical patella dislocation was first reported by Cooper *et al* in 1844, to our knowledge less than 40 cases have been reported until now. Patella rotation along its longitudinal axis with the articular surface facing laterally or medially is referred to as a vertical dislocation. When the rotated patella is locked inside the femoral groove, the dislocation is intra-articular; however, when the dislocated patella is wedged against the condyle, typically the lateral side of the lateral condyle, it is extra-articular. The patellar vertical dislocation that occurs extra-articularly is uncommon. According to other studies, the reduction was performed in a close or open reduction under general anaesthesia^1,2^.

The mechanism of vertical patellar dislocation is still unknown. Several mechanisms were proposed although they were not proven yet. This article delivers a case of a vertical patellar dislocation in an adolescent, which happened due to a low-energy injury, making this case a rare case to be presented.

## Case Report

This is a rare case presentation of a sixteen-year-old girl who suffered from a closed vertical dislocation of her left patella caused by a very low-energy injury. The mode of injury was due to the patient leaning on her knee as she climbed onto the bed. She felt pain and a twisted feeling in the left knee during this event. She claimed a "clicking" sensation in her left knee. Then, her left knee was suddenly unable to move.

**Fig 1:**
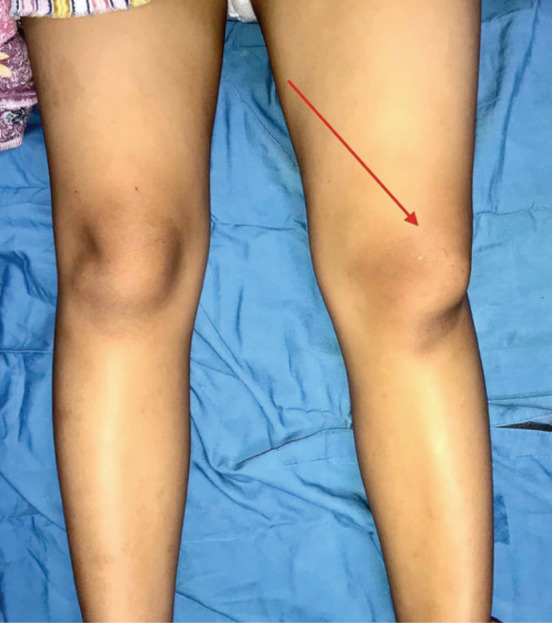
Initial clinical presentation showing dorsal fin appearance as shown by the red arrow.

Upon physical examination, we found a deformity and skin tenting, with the characteristic dorsal fin appearance over the laterally displaced patella and tenderness on palpating the medial and superior sides of the patella. The patella was malpositioned, and the patella’s articular surface was rotated and facing toward the lateral side (Fig. 1).

**Fig 2:**
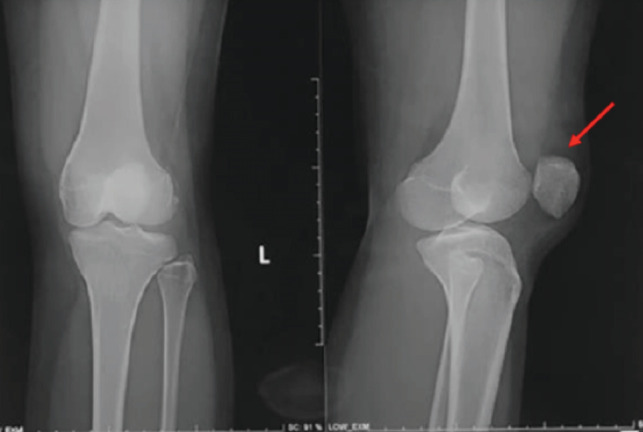
Initial plain radiograph evaluation showing a malposition of the patella with 90° of rotation on the vertical axis as shown by the red arrow.

We evaluated the presence of general laxity by assessing the clinical features of this patient using Beighton’s score, and the total score is 4. Initial plain radiograph evaluation shows malposition of the patella with 90º rotation on the vertical axis as shown by the red arrow (Fig. 2).

**Fig 3:**
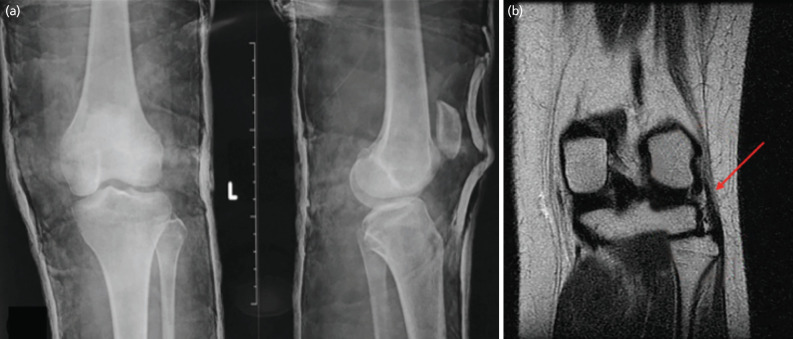
(a) Post-reduction radiograph. (b) MRI showed partial discontinuity of the left lateral collateral ligament and joint effusion. No osteochondral defect or loose bodies (shown by the red arrow) existed.

Under general anaesthesia, closed reduction was performed, by pressing the patellar inferolateral pole superiorly and medially toward the flat part of the medial femur condyle by using the thumb until the patella was reduced. Following the reduction, immobilisation with a circular cast was maintained for three weeks. The reduction was evaluated using post-reduction radiograph (Fig 3a).

The patient’s left knee soft tissues were examined using MRI evaluation done two days following the reduction. MRI showed partial discontinuity of the left lateral collateral ligament and joint effusion. There was no osteochondral defect and loose bodies (Fig. 3b).

We removed the cast after three weeks, and a physical examination showed the patient able to move her knee flexion up to 140°, and the patient was referred for physical rehab. After the 6th week of follow-up, the patient achieved a full and painless range of motion, and there was no reported complaint. The functional score using Kujala score in 3rd week, 6th week, and 12th week are 52%, 75%, and 97%, respectively.

## Discussion

Patella dislocation is an emergency with annual incidence of 5.8 per 100,000 individuals. Cooper et al initially described a new case and its details about vertical patellar dislocation. Until 1978, less than 40 cases were reported internationally. Acute dislocation of the patella is most commonly caused by direct impact or a twisting mechanism during physical activities^1,3^.

Dislocation of the patella can be treated with closed reduction. If closed reduction cannot be achieved under conscious sedation, treatment under general anaesthesia is suggested^2^. In this case, the patient is treated with closed reduction under general anaesthesia followed by immobilisation with a circular cast for three weeks as performed by Ahmad Khan *et al*^3^. Another case was reported which was treated with open reduction using reduction clamp and immobilised in a plaster cast for six weeks, another case was reported which was treated with open reduction using reduction clamp and immobilised in a plaster cast for six weeks. In approximately eight weeks after the injury, the patient recovered to normal condition^2^.

In this case, the patient suffered a vertical patellar dislocation from a relatively low-energy injury. The patient also has generalised joint laxity. This is a very interesting case because it provides further information to the possibilities of the mode of injury and the risk factors of vertical patellar dislocation. Although vertical patellar dislocation is a rare case, it may occur in adolescent patient and those with generalised joint laxity^3-5^. Three similar cases were reported, in adolescent patients with generalised joint laxity who suffer minor trauma, resulting in vertical patellar dislocation^1,2,5^.

In contrast to open reduction that was documented in the literature, the described case was distinctive in that a closed reduction was accomplished while the patient was under general anaesthesia. The extensor was relaxed while the quadriceps were contracted toward the knee. The lateral condyle's prominent portion could be avoided since the patellar was lifted and pushed cranial-medially with the knee in hyperextension. In situations like these, it is suggested that closed reduction under general anaesthetic be tried^1,2^.
